# Reasons why specialist doctors undertake rural outreach services: an Australian cross-sectional study

**DOI:** 10.1186/s12960-016-0174-z

**Published:** 2017-01-07

**Authors:** Belinda G. O’Sullivan, Matthew R. McGrail, Johannes U. Stoelwinder

**Affiliations:** 1 Monash Rural Health, Office of Research, Level 3, 26 Mercy St, PO Box 666, Bendigo, Victoria 3550 Australia; 2 Monash Rural Health, Northways Road, Churchill, Victoria 3842 Australia; 3 Division of Health Services and Global Health Research, School of Public Health and Preventive Medicine, Monash University, Melbourne, Victoria 3004 Australia

**Keywords:** Specialist doctor, Outreach, Reasons, Services, Rural

## Abstract

**Background:**

The purpose of the study is to explore the reasons why specialist doctors travel to provide regular rural outreach services, and whether reasons relate to (1) salaried or private fee-for-service practice and (2) providing rural outreach services in more remote locations.

**Methods:**

A national cross-sectional study of specialist doctors from the Medicine in Australia: Balancing Employment and Life (MABEL) survey in 2014 was implemented. Specialists providing rural outreach services self-reported on a 5-point scale their level of agreement with five reasons for participating. Chi-squared analysis tested association between agreement and variables of interest.

**Results:**

Of 567 specialists undertaking rural outreach services, reasons for participating include to grow the practice (54%), maintain a regional connection (26%), provide complex healthcare (18%), healthcare for disadvantaged people (12%) and support rural staff (6%). Salaried specialists more commonly participated to grow the practice compared with specialists in fee-for-service practice (68 vs 49%). This reason was also related to travelling further and providing outreach services in outer regional/remote locations. Private fee-for-service specialists more commonly undertook outreach services to provide complex healthcare (22 vs 14%).

**Conclusions:**

Specialist doctors undertake rural outreach services for a range of reasons, mainly to complement the growth and diversity of their main practice or maintain a regional connection. Structuring rural outreach around the specialist’s main practice is likely to support participation and improve service distribution.

## Background

Universal health coverage is integral to the sustainable development goals of the United Nations, to promote healthy lives and promote well-being for all at all ages. However, developing and developed countries [1–7] alike face significant challenges ensuring adequate access to specialist medical care in rural areas, where it is most needed. One of the causes of maldistribution of specialist services is that they are often not viable in rural communities on a full-time basis. Yet access to local specialists is important to contribute to the management of more complex and severe illnesses in rural areas, mitigating out-of-community referral and can improve continuity of care [8]. Various service models are used around the globe to increase access to local specialists, including outreach or visiting consultant models, where specialists travel away from their main practice to provide regular services in a rural location [1, 9–11].

Rural outreach participation by specialists is a potentially scalable policy strategy. It is relatively common, at least in developed countries, where there are limited studies alluding to its prevalence. An estimated 58% of Iowa-based urologists [12] and half of oncologists [13] provide visiting consultancy services to rural areas. In Australia, about 19% of all specialists undertake rural outreach work, varying by specialist type (42–44% of urologists and renal physicians, 30–33% oncologists and otolaryngologists and as low as 13% by sub-specialist surgeons) [14].

The reasons why specialists undertake rural outreach work have not been systematically explored, despite being integral to planning policy and programs that align with workforce interests. Altruism underpinning international “medical missions” into poorer countries is considered as having the potential to drive more rural outreach services by otolaryngologists in the USA [6]. However, there is limited systematic evidence to assess the prevalence of altruism. Over-emphasising altruism could deflect attention from system-based influences that are amenable to policy intervention.

The only other study about reasons for rural outreach work comes from a US survey of specialists visiting 11 rural hospitals in Massachusetts, who identified supplementing the patient base and income and supporting underserved patients, although such reasons only applied to 25% of all respondents [15]. Further evidence of reasons can be deducted from multiple case reports that suggest specialists participate in outreach to provide equitable healthcare [16–18], support local workers [19], maintain a connection to a region [20], undertake interesting medicine [21] and capture an increased market share of patients [18, 22]. The prevalence of such reasons at a national level has never been systematically measured.

This study explores the reasons why specialist doctors travel to provide regular rural outreach services, and whether reasons relate to (1) salaried or fee-for-service practice and (2) providing outreach services in more remote locations.

## Methods

### Sample

A cohort of specialist doctors, working clinically and travelling away from their main practice, to provide regular, rural outreach services was identified in a large national longitudinal panel survey of doctors, the Medicine in Australia: Balancing Employment and Life (MABEL) study. The primary aim of the MABEL study is to investigate the labour supply decisions and their determinants among Australian doctors. The study protocol has been reported elsewhere [23], but briefly, beginning in 2008, between June and November, all Australian doctors working clinically (*n* = 54,750) were invited to participate. Contact details were obtained from Australia’s Medical Publishing Company [24], considered the most comprehensive and accurate listing of all national medical practitioners available at the time. Doctors (general practitioners, specialists, specialists in training and hospital non-specialists) were sent an invitation and study information, a paper copy of the survey, and were given the opportunity to complete the survey online through a secure website, with three reminders issued. Specialist doctors had completed advanced medical training to gain a fellowship with a specialist college (excluding family physicians) [25]. The survey collected information about personal characteristics, employment conditions, finances, geographic location and outreach work.

In 2008, a total of 10,498 doctors responded, including 4310 specialists (22% response rate). Every subsequent year, all respondents from previous years are re-surveyed along with new doctors, (returning to active clinical practice, recent immigrants or new graduates) between June and November. We report the results of 3505 specialist doctors responding to the survey in 2014, being the first year the survey included more detailed questions of specialists participating in rural outreach. Outreach providers were identified as specialists reporting (1) “yes” to a question “do you travel to provide services/clinics in other geographic areas?” and listing up to three locations (town/suburb) and postcodes (away from the main practice), which were geocoded to test rurality and (2) reporting they provided this service on a regular, periodic basis (an “outreach” service). The 2014 questionnaire can be accessed from http://mabel.org.au/research/questionnaire/wave7.

Analysis of non-response bias (based on key covariates of age, sex, geographic location, doctor type and hours worked) specific to the first 2 years of the MABEL survey (2008–2009) has been reported elsewhere, showing the responding cohort was broadly representative of all Australian doctors [23, 26]. Specific to the 2014 cohort, (*n =* 3505), Table 1 shows respondents were comparable to the national specialist workforce but had 8% more females, a lower mean age (45 vs 50 years) and around 5% fewer surgeons.

**Table 1:** Characteristics of respondents to the Medicine in Australia: Balancing Employment and Life survey, 2014, compared with the Australian specialist workforce

	Specialist respondents (*n =* 3505)		Australian specialist workforce (*n =* 27,279)^c^	
	*n*	%	*n*	%
Sex				
Male	2260	65	19,681	72
Female	1243	36	7598	28
Mean age (years)^a^	45		50	
Location main work				
Metropolitan	2899	83	21,808	86
Rural	606	17	3601	14
Specialist group^b^				
Internal medicine	762	22	5706	21
Pathology	127	4	1119	4
Surgery	380	11	4250	16
Other specialists	1986	57	15,306	56
Mean hours worked/week	42		44	

^a^The number of sample respondents to *age was* reduced to 3441 due to 64 missing values; *sex* reduced to 3503 due to 2 missing values; *mean hours worked* reduced to 3239 due to 266 missing values and *specialist group* reduced to 3255 due to 250 missing values

^b^Internal medicine: cardiology, endocrinology, gastroenterology and hepatology, general medicine, geriatric medicine, haematology, medical oncology, nephrology, respiratory and sleep medicine, rheumatology, other physician. Pathology: anatomical and general pathology. Surgery: general surgery, otolaryngology, plastic, urology, other surgery. Other specialists: diagnostic radiology, other radiology, obstetrics and gynaecology, paediatrics, anaesthesia, psychiatry, emergency medicine, ophthalmology, dermatology, intensive care medicine, rehabilitation medicine, radiation oncology, other specialists not grouped

^c^Data on the Australian specialist workforce were obtained from the National Health Workforce Dataset (NHWDS), 2014 [36], except data on *Location main place of work*, which was obtained from the 2014 Australian Medical Directory dataset (*n =* 25,409) [24]. The NHWDS included *n =* 166 specialists whose specialty was general practice under “other specialists”, which is not included as a specialty in the MABEL survey. Further 898 in the NHWDS dataset were missing information about their specialist group

### Australian context

Australia is a large country with notable health disparities between metropolitan and regional and remote populations. Around a third (30%) of Australians live in rural areas, where only 15% medical specialists work [27, 28] (Table 2). The availability of local specialist services diminishes with population remoteness (152.8 per 100,000 population in metropolitan areas, compared to 78.8, 58.2 and 33.0 in inner regional, outer regional and remote areas, respectively) [28]. Like other countries, complex co-morbid illness is more common in rural and remote populations because the lack of local services and long distances to reach service centres tends to delay medical intervention and limit the amount of follow-up care individuals receive. Indigenous Australians are over-represented in rural and particularly remote areas and their access to mainstream services is also affected by cultural and financial barriers [11]. The rates of trachoma [29], otitis media [19] and rheumatic heart disease [16] among indigenous Australians in remote communities remain high relative to global expectations.

**Table 2:** Geographic properties of the Australian Statistical Geography Standard – Remoteness Area (ASGS-RA) categories [27, 31]

ASGS-RA	Label	Australia’s population (%)	Australia’s area %	Density (persons per km^2^)
1	Major city	69.9	0.2	794
2	Inner regional	18.6	3.2	16.2
3	Outer regional	9.2	10.2	2.5
4	Remote	1.4	12.0	0.3
5	Very remote	0.9	74.4	0.03

Around two thirds of Australian specialists work on a fee-for-service basis in private consulting rooms or private hospitals [30]. The financial viability of their practice depends on adequate clinical caseloads. They are reimbursed for clinical services through a national health payment scheme (Medicare) and the patient’s voluntary private health insurance and co-payment.

In contrast, specialists employed on salaries are commonly based in large public (government-funded) hospitals. Their remuneration is not affected by time spent travelling, and rural outreach service roles are often embedded in their employment requirements and factored into their job choice [30].

It is possible that salaried specialists participate in rural outreach for different reasons to those in private fee-for-service practice, who are potentially more driven by financial considerations.

### Outcome variables

The outcome variables were the reasons specialists participated in rural outreach work, identified from a series of questions that were developed and added to the 2014 MABEL survey. The range of reasons was based on those identified within published case studies and was deliberately diverse to draw out various planning implications, including the prevalence of altruism. All questions were closed-ended to fit with other questions in the MABEL survey and ensure consistency via self-reported methods. A limited number of reasons could be included, based on the length of the MABEL survey. The questions were piloted in two stages, firstly by checking the scope and clarity among a group of ten specialists providing rural outreach services, to which four responded. Based on the feedback, the reason: “supporting rural health staff” was added. Secondly, by formally piloting them within the 2014 MABEL survey, which was sent to a random sample of 150 specialists, between January and March 2014. Minimal refinement was needed before the questions were included in the final survey.

Specialists travelling away from their main practice to provide regular rural outreach services in the last year, reported their agreement on a 5-point scale with five reasons for providing their main outreach service (where they spent the most time): “I provide this service in order to (1) grow my practice, (2) provide healthcare to disadvantaged people, (3) maintain a personal connection to a region, (4) provide complex healthcare in challenging situations and (5) provide support for rural health staff”. Responses were categorised: agree (strongly agree/agree) or do not agree (neutral/disagree/strongly disagree). Based on the Australian Statistical Geography Standard-Remoteness Area (ASGS-RA) categories of the specialist’s town and postcode of residence, location was categorised into two groups: “metropolitan” (ASGS-RA 1) or “rural” (ASGS-RA 2 to ASGS-RA 5) which encompassed inner regional, outer regional and remote/very remote locations [31]. The geographic properties of this scale are outlined in Table 2.

### Measuring other variables

To measure the extent of rural outreach work undertaken as a requirement of the specialist’s main employment, a new question was also piloted and added to the 2014 MABEL survey: “Are you required to provide outreach services as part of your employment conditions at your main place of work?”

The average weekly hours worked in public or private hospitals, private consulting rooms or other settings were an existing variable of the annual MABEL survey and were used to define salaried (all hours in public hospitals) or private fee-for-service practice (some hours in private hospitals or consulting rooms).

The main rural outreach service location (already geocoded according to postcode of service location) was further categorised as: “inner regional” or “outer regional and remote” defined as levels 2 and levels 3–5, respectively, on the ASGS-RA scale [31] (Table 2). Another new question added to the 2014 MABEL survey asked specialists participating in rural outreach work, the travel time to reach the location of their main outreach service from their place of residence: “<1 h”; “1–3 h”; or “4+ h”.

### Statistical analysis

The association between agreement with the reasons for participating, whether specialists work in salaried or private fee-for-service practice, and service characteristics was tested using Pearson chi-squared with a significance level of 5%. For the analysis of main employment, 37 specialists were excluded because their main practice was not based in hospitals or consulting rooms, rather areas like laboratories and academia. Statistical analysis was performed with STATA LC 11.2 [32].

## Results

Of the 3505 specialist doctors responding to the 2014 MABEL survey, 645 provided a rural outreach service (18%). Within this group, 45 were excluded because their main outreach service location was indeterminate, 25 were missing information about reasons for providing the service and 8 were neutral to all reasons, leaving 567 in the final cohort. No exclusion bias was detected by age (*P* = .28) or sex (*P* = .07).

Around half of the specialists (54%) reported undertaking their main rural outreach service to grow the practice; 26% to maintain a personal connection to a region; and 18% to provide complex healthcare in challenging situations (Table 3). Less commonly, specialists reported participation in outreach to provide healthcare to disadvantaged people (12%) and support rural health staff (6%).

**Table 3:** Association between covariates and reasons specialist doctors undertake rural outreach services, based on Pearson chi-squared

	Agreement with reasons											
Covariate			Grow my practice		Maintain personal connection to region		Complex healthcare in challenging situations		Provide healthcare for disadvantaged people		Provide support rural staff	
			*n* (%)	*P*	*n* (%)	*P*	*n* (%)	*P*	*n*	*P*	*n* (%)	*P*
		*n*	304 (54)		145 (26)		104 (18)		70 (12)		35 (6)	
Location	Metropolitan	385	206 (55)		112 (30)		68 (18)		50 (13)		27 (7)	
	Rural	180	97 (54)	.87	32 (18)	.003	35 (20)	.65	19 (11)	.39	8 (4)	.22
Main practice	Salaried only	196	127 (68)		56 (29)		27 (14)		28 (14)		7 (4)	
	Fee-for-service	332	158 (48)	<.0001	80 (24)	.22	72 (22)	.027	35 (11)	.20	25 (8)	.07
Outreach location	Inner regional	340	163 (49)		96 (29)		73 (22)		53 (16)		27 (8)	
	Outer regional/remote	227	141 (64)	.001	49 (22)	.079	31 (14)	.017	17 (8)	.004	8 (4)	.031
Time travelled	<1 h	86	39 (47)		18 (21)		24 (29)		14 (16)		9 (10)	
	From 1–3 h	342	178 (53)		92 (27)		60 (18)		41 (12)		17 (5)	
	4+ h	137	86 (66)	.009	34 (26)	.56	19 (14)	.022	14 (10)	.41	9 (7)	.17
Missing			14 (3)		11 (2)		7 (1)		7 (1)		5 (1)	

Metropolitan-based specialists were more likely than rural specialists to provide the main outreach service to maintain a connection to a region (30 vs 18%, *P* = .003) (Table 3).

Around a quarter of specialists were required to undertake rural outreach services as part of their normal work (26%), related to salaried practice compared to private fee-for-service practice (40 vs 14%, *P* < .0001). Salaried specialists also more commonly reported providing the outreach service to grow the practice compared with private fee-for-service specialists (68 vs 48%, *P* < .0001) (Table 3).

Growing the practice was additionally related to providing outreach services in an outer regional or remote town (64 vs 49%, *P* = .001) and travelling for longer (66 vs 47%, *P* = .009) (Table 3).

Specialists in a private fee-for-service practice more commonly participated to provide complex healthcare in challenging situations (22 vs 14%, *P* = .027), which was also associated with providing outreach services to an inner regional location (29 vs 14%, *P* = .017) and travelling less time (29 vs 14%, *P* = .022) (Table 3).

## Discussion

Specialist doctors travel to provide regular rural outreach services for a range of reasons, primarily related to supporting the growth and diversity of their main practice. Growing the practice was reported by half the cohort, followed by other key reasons of maintaining a personal connection to a region and providing complex healthcare in challenging situations. Only around 5–10% specialists reported participating to provide healthcare to disadvantaged people and supporting rural health staff, which suggests altruistic reasons are secondary to professional interests for those participating. Other research about doctor’s choosing to practice in small rural towns has suggested that community involvement and self-actualisation are among four key motivators [33]. However, outreach work is usually auxiliary to the main practice, and whilst altruism may inform the decision to participate, interests and commitments to the main practice are likely to play a more pivotal role in shaping participation.

Our study extends on the single-state survey by Drew et al. [15] by exploring how reasons vary according to the nature of the specialist’s practice at a national level. Salaried specialists were more likely to report outreach to grow the practice. Growing the practice is easier to describe as an interest for specialists in private fee-for-service practice, where revenue from service volume is directly paid to the specialist. It is somewhat more complex to explain for salaried specialists with a set income where reasons beyond financial gain must be at play. It potentially reflects the goals of public healthcare to increase access to specialist services among targeted, disadvantaged groups, in more outer regional and remote locations, based on the longer distances travelled by specialists stating this reason. There are several examples of state-wide outreach programs, involving highly specialist teams, regularly travelling from large metropolitan public hospitals, to target Indigenous health priorities [16, 34]. Apart from increasing the range of patients accessing the service, outreach into Indigenous communities is likely to extend the specialist’s cultural dimensions of practice.

Alternatively “growing the practice” may reflect a complex array of professional and system-level competition among salaried specialists linked to their hospital employment. Professionally, rural outreach work could develop relationships and partnerships in underserved communities, potentially tapping into new service areas. This is important if salaried specialists wish to transition to more private practice or want to build professional status to achieve career advancement as a staff specialist within their employing hospital. Also, public hospitals typically limit the number of procedures/clinics available to employed specialists due to budget restrictions, increasing the potential for salaried specialists to periodically travel to smaller under-utilised hospitals to increase patient throughput.

Undertaking rural outreach work to provide complex healthcare in challenging situations, more common among specialists in private fee-for-service practice, is likely to be related to broadening the scope of work outside of the main practice. To generate revenue, specialists in private fee-for-service practice may be inclined to focus their main practice on relatively uncomplicated, higher prevalence conditions. Outreach work potentially provides the opportunity for a unique and intellectually stimulating caseload, enabling blended practice experiences [21]. Specialists interested in complex healthcare in challenging situations did not travel as far and had a higher rate of outreach services provided in inner regional locations. Potentially, goals for practice diversity are achievable without travelling far from the main practice base. The scope of technical practice is likely to be wider if specialists visit larger rural hospitals with more infrastructure and support staff, as opposed to delivering services in primary care settings that are often the only option in more remote contexts. This is particularly relevant to practising procedures that require sterile conditions. Alternatively, specialists in fee-for-service practice with interests in complex healthcare need to balance such goals with the financial viability of their outreach service. Rural specialists in private fee-for-service practice tend to travel shorter distances to provide rural outreach services than equivalent specialists in metropolitan areas probably because they have less efficient travel options available [35].

Finally, this study shows that metropolitan and rural-based specialists undertake rural outreach for similar reasons, noting one exception. More metropolitan-based specialists participated to maintain a personal connection to a region, suggesting rural exposure during training or work could increase outreach participation. Previous research noted childhood rural background was not a factor influencing rural outreach work [14]; however, it is possible that other childhood exposures, such as visiting rural relatives or rural holidays could be relevant.

### Strengths and limitations

Although this study is based on a national, relatively unbiased cohort and accounts for a higher proportion of all specialist reasons than the one other study in this field [15], it was limited in scope. It only explored five reasons using closed-ended questions, within a self-reported survey. Whilst an attempt was made to explore key reasons that were evident from existing literature, the list of reasons was not exhaustive; 51 specialists participating in rural outreach disagreed or were neutral to all the reasons listed.

As a cross-sectional survey, the results are limited to associations, rather than causality, and do not account for the possibility that reasons for providing a rural outreach service could vary within individuals over time and by their career stage. However, with regard to career stage, our data indicated no differences between the reasons of specialists in early (<45 years) versus mid/later career stages (45+). The number of statistical tests performed could have increased the risk of type I errors.

The reasons for participating were only explored in relation to the specialist’s main rural outreach service, whereas specialists could be providing more than one outreach service, visiting different towns for different reasons.

This study raises questions about how specialists remunerated in different ways interpret “growing the practice”, providing the basis for further research. The reasons confer with the key published US study by Drew et al. [15], but more specifically qualify that compared with altruistic drive, specialists are interested in outreach work to complement their main practice.

Further work is needed to clarify how well these reasons might translate to other countries, respecting the varied practice context and remuneration patterns of specialist doctors related to their national context. Further, it would be worth considering whether reasons for participating in outreach healthcare at a national level are similar to reasons underpinning international outreach work.

## Conclusions

Specialist doctors undertake rural outreach work for a range of reasons, mainly to support the growth and diversity of their main practice, rather than to support disadvantaged populations or rural health staff. Specialists in salaried practice more commonly undertake rural outreach services to grow the practice. Private fee-for-service specialists were more likely to participate so as to provide complex healthcare in challenging situations. Structuring rural outreach around the specialist’s main practice is likely to support participation and improve service distribution.
